# Comparative liver transcriptome analysis in hamsters infected with food-borne trematodes *Opisthorchis felineus*, *Opisthorchis viverrini*, or *Clonorchis sinensis*

**DOI:** 10.1371/journal.pntd.0012685

**Published:** 2024-12-09

**Authors:** Ekaterina A. Lishai, Oxana G. Zaparina, Yaroslav K. Kapushchak, Banchob Sripa, Sun-Jong Hong, Guofeng Cheng, Maria Y. Pakharukova

**Affiliations:** 1 Institute of Cytology and Genetics, Siberian Branch of Russian Academy of Sciences (ICG SB RAS), Novosibirsk, Russia; 2 Department of Natural Sciences, Novosibirsk State University, Novosibirsk, Russia; 3 Chung-Ang University College of Medicine, Seoul, Korea; 4 WHO Collaborating Centre for Research and Control of Opisthorchiasis (Southeast Asian Liver Fluke Disease), Tropical Disease Research Center, Department of Pathology, Faculty of Medicine, Khon Kaen University, Khon Kaen, Thailand; 5 Shanghai Tenth People’s Hospital, Institute for Infectious Diseases and Vaccine Development, Tongji University School of Medicine, Shanghai, China; Beijing Friendship Hospital, Capital Medical University, CHINA

## Abstract

**Background:**

Epidemiologically important food-borne trematodes *Opisthorchis viverrini* and *Clonorchis sinensis* are recognized as biological carcinogens of Group 1A, while *Opisthorchis felineus* is in Group 3 as noncarcinogenic to humans. Mechanisms of the biological carcinogenesis are still elusive. Some studies highlight chronic inflammation as a key factor and common pathway for cancer initiation and progression. Nonetheless, the chronic inflammation alone does not explain why these three species differ in carcinogenicity. We focused this study on genome-wide landscapes of liver gene expression and activation of cellular pathways in *Mesocricetus auratus* golden hamsters infected with *C*. *sinensis* (South Korea), *O*. *viverrini* (Thailand), or *O*. *felineus* (Russia) at 1 and 3 months after infection initiation.

**Methodology/Principal findings:**

Liver transcriptomes of golden hamsters (HiSeq Illumina, 2X150 bp) were sequenced at 1 and 3 months postinfection. Data processing was carried out using the following bioinformatic and experimental approaches: analysis of differential gene expression, estimates of proportions of affected liver cell types, liver histopathology, and examination of weighted gene coexpression networks. All infections caused enrichment with inflammatory response signaling pathways, fibrogenesis and cell proliferation, and IL2–STAT5, TNF–NF-κB, TGF-β, Hippo, MAPK, and PI3K–Akt signaling pathways. Nevertheless, species-specific responses to each infection were noted too. We also identified species-specific responses of liver cell types, differentially expressed gene clusters, and cellular pathways associated with structural liver damage (such as periductal fibrosis, epithelial neoplasia, and inflammation).

**Conclusions/Significance:**

This is the first comparative analysis of gene expression landscapes in the liver of experimental animals infected with *O*. *viverrini*, *O*. *felineus*, or *C*. *sinensis*. The trematodes have species-specific effects on the hepatobiliary system by triggering signaling pathways, thereby leading to differences in the severity of hepatobiliary structural lesions and contributing to the pathogenicity of closely related foodborne trematodes.

## Author summary

Food-borne trematodiases constitute an important group of the neglected tropical diseases caused by *Opisthorchis felineus*, *O*. *viverrini*, and *Clonorchis sinensis* liver fluke infections. These parasites affect the hepatobiliary system of mammals, including humans, by inducing cholangitis, bile duct neoplasia, and even cholangiocarcinoma in chronically infected individuals. Two species, *O*. *viverrini* and *C*. *sinensis*, are recognized group 1A biological carcinogens to humans, whereas *O*. *felineus* is classified as a noncarcinogen. Mechanisms of formation of the cholangiocarcinoma associated with helminth infection remain unknown. We focused on the research into early events that lead to precancerous changes. Here we examined differences in genome-wide profiles of liver gene expression and activation of cellular pathways in *Mesocricetus auratus* hamsters infected with *C*. *sinensis* (South Korea), *O*. *viverrini* (Thailand), or *O*. *felineus* (Russia) at 1 and 3 months after infection initiation. Infections with *C*. *sinensis* and *O*. *felineus* mostly featured development of periductal fibrosis accompanied by a huge inflammatory response in the liver. *O*. *viverrini* infection, however, showed severer hyperplasia and biliary-intraepithelial-neoplasia lesions. Thus, these related food-borne trematodes have species-specific effects on the liver during infection, which likely contribute to the differences in carcinogenic potential among these trematodes.

## 1. Introduction

Neglected infectious diseases caused by the food-borne trematodes *Opisthorchis viverrini*, *Opisthorchis felineus*, and *Clonorchis sinensis* afflict populations not only in the tropical regions of East Asia, but also in temperate and semi arctic areas of Europe and Asia. Long-lasting infections with these helminths are associated with chronic inflammation and lead to hepatic periductal and cholangiofibrosis, biliary intraepithelial neoplastic changes, and a bile duct cancer: cholangiocarcinoma [1–4]. The prognosis of patients with this type of cancer remains very poor: the 5-year survival rate ranges from 15% to 25%, and in the presence of metastases drops to 2% [5].

**Fig 1:**
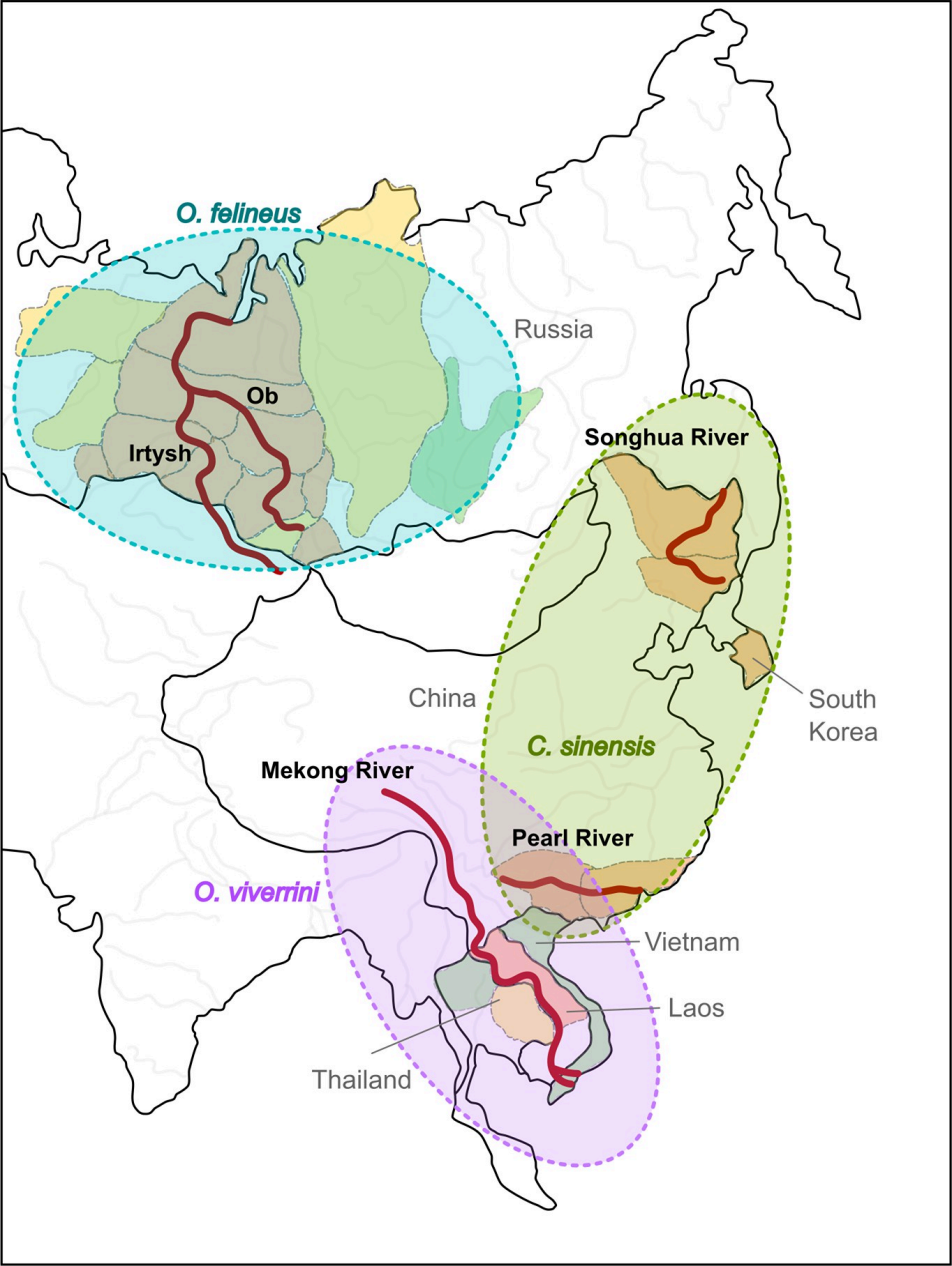
Highly endemic areas of food-borne Opisthorchiidae liver flukes. The image was created by Lishai EA with the use of a basemap from the stock of free vector maps https://www.sharada.ru/pdf-maps/maps/kontinenty-i-chasti-sveta/konturnaja-karta-evrazii.

Differences among the three liver flukes concern the number of chromosomes in the karyotypes of these species, in particular, *O*. *felineus* and *C*. *sinensis* have seven pairs of chromosomes (2n = 14), while *O*. *viverrini* has six pairs (2n = 12) [6]. Geographic ranges of the three species are also different and do not overlap (Fig 1). For instance, *O*. *viverrini* is endemic to Southeast Asia [7]; *O*. *felineus* infection is typically registered in Russia and some European countries [reviewed by 1]; and *C*. *sinensis* infection in China and South Korea [8]. The most striking difference is carcinogenicity to humans. In particular, *O*. *viverrini* and *C*. *sinensis* are recognized as Group 1 biological carcinogens, while *O*. *felineus* is classified as a Group 3 agent: noncarcinogenic to humans [9]. This observation implies dissimilarity of the mechanisms of bile duct epithelium malignant transformation induced by these liver flukes.

Studies indirectly indicate different activated liver signaling pathways during infection with the different liver flukes. For instance, activation of immune response genes in the mouse liver was revealed at the stage of early infection, whereas a decrease in the activity of these genes at 6 weeks after *C*. *sinensis* infection initiation. In addition, the expression of genes involved in lipid metabolism is suppressed during such an infection, whereas Wnt signaling proteins (Wnt7b, Fzd6, and Pdgfrb) and cell cycle–regulatory proteins (cyclin D1, Cdca3, and Bcl3) are upregulated [10]. Findings consistent with these results were obtained in a study on *C*. *sinensis* infection in rats [11]. Data on *O*. *felineus* infection in hamsters show that different cellular pathways are activated during acute infection and during chronic infection; the most enriched pathway during this infection is epithelial–mesenchymal transition [12]. In another work, after *O*. *viverrini* infection initiation, RB pathway genes were investigated in liver cells of hamsters; the researchers revealed that the expression of genes *RB1* and *p16INK4* decreased, whereas the expression of cyclin D1 and cyclin-dependent kinase CDK4 genes increased. Similar results have also been obtained in humans [3].

A comparison of three closely related species with different carcer risks appears to be a promising model for identifying key processes of liver metabolic reprogramming during biological carcinogenesis and for uncovering the cascade of regulatory events and dynamics of precancerous changes.

Therefore, it is difficult to describe the entire scheme of changes in gene expression and in cellular pathways under the influence of trematode infection and to detect the differences that could be key precancerous events. This is because the studies have been conducted via different sequencing approaches on different species of laboratory animals. Furthermore, carcinogenic potential may be determined not only by differences in the genome and biology among the liver flukes themselves but also by national features of human nutrition, ecology, climate, environmental pathogens, and/or microbiota. A direct comparison of liver transcriptomic landscapes to the three liver fluke infections has not been reported.

The aim of this study was to identify genome-wide gene expression profiles and activation of cellular pathways in liver cells of *Mesocricetus auratus* hamsters infected with one of the trematode species: *O*. *viverrini*, *O*. *felineus*, or *C*. *sinensis*. The work was also focused on estimation of proportions of affected liver cell types in bulk expression data as well as identification of clusters of differentially expressed genes (DEGs) and cellular pathways associated with structural liver lesions.

## 2. Methods

### 2.1. Ethics statement

All the procedures were in compliance with EU Directive 2010/63/EU for animal experiments. Study design protocols and standard operating procedures (concerning the hamsters and the fish) were approved by the Committee on the Ethics of Animal Experiments at the ICG SB RAS (permit number 42 of 25 May 2018).

### 2.2. Experimental design

Twenty-four SPF (specific pathogen-free) golden Syrian hamsters were used in this study. All the procedures with hamsters were performed at the SPF animal facility of the ICG SB RAS. For collecting *O*. *felineus* metacercariae, a naturally infected freshwater fish (*Leuciscus idus*) was net-caught in the Ob River near Novosibirsk (Western Siberia, Russia). *O*. *felineus* metacercariae were extracted as described previously [13]. *C*. *sinensis* and *O*. *viverrini* metacercariae were extracted from naturally infected freshwater fish (Seoul, Republic of Korea, and Khon Kaen, Thailand, respectively). The extracted metacercariae were identified under a light microscope.

Two-month-old male hamsters (*M*. *auratus)* were randomly subdivided into four groups: uninfected (group 1) and infected orally with 75 metacercariae of *O*. *felineus* (2), *C*. *sinensis* (3), or *O*. *viverrini* (4) by gastric intubation. One and 3 months after the infection initiation (postinfection: p.i.), three hamsters from the uninfected group and three hamsters from each of the other groups were euthanized using carbon dioxide. All the infections were conducted with a difference of approximately 5–7 days between infections with each species of trematode. All hamsters of the same age from different litters were combined into one group, from which hamsters were randomly selected for both the infected and uninfected groups to exclude the batch effect. Thus, the animals were also sacrificed with a difference of 5–7 days.

All the hamsters were examined daily for signs of illness, injury, or abnormal behavior by SPF-trained personnel. Food and water availability and the macroenvironment (temperature, humidity, noise, light intensity, and cleanliness) were also evaluated daily. No unexpected deaths of hamsters were registered during this study. To analyze the infection intensity the EPG score (number of eggs per gram stool) was calculated at 3 months p.i.. No statistically significant differences in EPG score were found among groups. The data indicate that liver flukes infected hamsters with the similar extent.

### 2.3. Tissue collection and RNA extraction

The liver was quickly excised and frozen in liquid nitrogen and then stored at -80°C until analysis. RNA was extracted from the frozen tissue with the Aurum Total RNA Extraction Kit (Bio-Rad, USA). The total RNA was purified on Agencourt RNAClean XP beads (Beckman Coulter, Germany). The quality and quantity of the total RNA were evaluated on a NanoDrop 2000 spectrophotometer. The quality of samples for RNA-seq was assessed using Agilent 2100 Bioanalyzer and the Total RNA Nano Kit (Agilent Technologies, USA). Only samples with an RNA integrity number greater than 8.0 were chosen for gene expression analysis.

### 2.4. Library construction and sequencing

Blind coding was used during DNA library preparation. All the DNA libraries were constructed by the same personnel approximately within 1 week in accordance with the standard New England Biolabs protocol. Briefly, polyA-tailed mRNAs were purified from 1 μg of total RNA using the NEBNext Poly(A) mRNA Magnetic Isolation Module. Then, directional cDNA libraries were created by means of the NEBNext Ultra II Directional RNA Library Prep Kit for Illumina. Size selection of DNA fragments was performed on Agencourt AMPure XP beads (Beckman Coulter, USA). Next, PCR enrichment of the adapter-ligated library was conducted (six cycles of PCR). The size and quantity of the library were verified on the Agilent Bioanalyzer, and libraries were subjected to paired-end (2 × 150) sequencing on the Illumina HiSeq 2500 platform (Genewiz LLC, USA).

PCR enrichment of the adapter-ligated library was then performed (six PCR cycles). The size and quality of DNA libraries were verified using the Agilent High Sensitivity DNA Kit. The length of the target fragment in all libraries was ~320 bp.

### 2.5. Gene expression analysis

On average, ~37 million paired-end reads (range: 32–50 million) were obtained from each cDNA sample by Illumina stranded sequencing. The quality of raw sequencing data was checked using FastQC v.0.11.9, and the sequencing data were preprocessed with the Trimmomatic 0.36 tool to remove adapters and low-quality sequences [14]. The data were mapped to the *M*. *auratus* BCM_Maur_2.0 reference genome assembly in STAR version 2.7.10b [15]. To analyze differential gene expression, the Wald test with a threshold of 0.05 from R package DESeq2 (v.1.38.3) was performed [16]. The Benjamini–Hochberg correction for multiple testing was applied to the resulting p values, and the genes with an adjusted P value (P_adj_) < 0.05 and whose expression fold changes were greater than 2.0 were designated as DEGs. Weakly expressed genes (the sum of gene expression values for all libraries less than 47) were removed from the expression matrix.

Principal component analysis (PCA) for assessing the degree of library clustering was performed using the expression matrix of all genes after logarithmic transformation [function rlog() with parameter blind = F from R package DESeq2]. R package PCAtools (v.2.10.0) was employed for this analysis [17]

Pathway enrichment analysis was performed using Gene Ontology (GO), Kyoto Encyclopedia of Genes and Genomes (KEGG), and Molecular Signatures Database (MsigDB) databases based on mouse genome annotation [R package org.Mm.eg.db (v.3.16.3) for analysis via GO and KEGG, and R package msigdb (v.1.6.0) for analysis via MsigDB]. R package clusterProfiler (v.4.6.2) was used for this analysis [18,19].

Differences in the response of various cell types to infection were determined using R package BisqueRNA (v.1.0.5) and published data from single-cell RNA sequencing (scRNA-seq) analyses of the human liver [20,21].

Identification of the gene clusters manifesting specific patterns across samples was carried out by the likelihood ratio test (LRT) from the DESeq2 R-package and DEGreport (v.1.28.0) R-package [22]. Rlog-transformed normalized counts were employed to identify only DEGs (P_adj_ < 0.05), then the degPatterns function was executed to find the sets of genes that showed similar expression patterns across sample groups. Groups of genes from clusters were subjected to functional analysis to identify the associated functions.

Differences in the response of liver cell types to infection were evaluated by means of R package BisqueRNA (v.1.0.5) and published scRNA-seq data from the human liver [20,21]. The data distribution was checked for normality by the Shapiro–Wilk test and by construction of distribution histograms [stats (v.4.3.0) R package]. For normally distributed data, significance was tested by ANOVA followed by Tukey’s test for multiple comparisons. For data that were not normally distributed, the Kruskal–Wallis test was performed followed by the pairwise Kruskal–Wallis test with the Benjamini–Hochberg correction for multiple comparisons.

### 2.6. Histopathological analysis

For this analysis, the liver tissue from two lobes was carefully dissected, placed in 10% formaldehyde in phosphate-buffered saline (BioVitrum, Russia), and processed as described earlier [23–25]. The tissue slices were stained with hematoxylin and eosin by the standard method [23–25] and were examined under a light microscope (Axioskop 2 Plus; Zeiss, Germany).

To assess the severity of liver lesions, a semiquantitative histological analysis of the ratio (morphometry) type [26] was performed on all visual fields of two lobes of each animal. Blind coding was used during histopathology evaluation. The evaluation was conducted by two trained observers in parallel, and then a senior pathologist reviewed and advised on controversial cases. Sections of two liver lobes from each animal were stained with hematoxylin and eosin, after which a morphometric grid of 10 × 10 squares was superimposed on each consecutive field of view (100–150 photographs per animal). The number of squares occupied by such lesions as periductal fibrosis, cholangiofibrosis, infiltration by inflammatory cells, BiliN, and hyperplasia of the bile duct epithelium was determined, and the proportion of the liver area occupied by the lesion (P) was calculated using the formula: P = (X/Y) × 100%, where X is the number of affected squares, and Y is the number of all squares in all fields of view. Each field of view was scored, and a mean score was assigned to the whole tissue of that sample [24–26]. A heat map was constructed using the heatmap.2 (v.2.38) R package.

### 2.7. Weighted gene coexpression network analysis (WGCNA)

This analysis was carried out to search for clusters of coexpressed genes [27]. It allows to detect gene clusters having similar expression patterns and to calculate a correlation of these clusters with experimental conditions or clinical manifestations [27]. For the analysis, we chose genes for which P_adj_ < 0.05 in at least one type of infection (9557 genes). The Pearson correlation coefficient was used to assess the correlation. An optimal threshold value was selected between 1 and 20 using the pickSoftThreshold() function with parameters RsquaredCut = 0.8 and networkType = "signed hybrid." A calculated threshold value of 14 was utilized for this study (WGCNA R package version 1.72–5).

The correlation of clusters with various structural liver lesions (periductal fibrosis, inflammation, cholangiofibrosis, and dysplasia) was computed from the semiquantitative histopathological data (stats R package version 4.3.0). Analysis of functional enrichment of clusters was carried out with R packages clusterProfiler and msigdb in the MSigDB database.

### 2.8. Western blotting

Liver lysates were prepared using RIPA Buffer according to a previously described method [23]. The protein concentration in each lysate was determined with the BCA Protein Assay Kit (Thermo Scientific, USA). Western blot analysis was performed in accordance with the standard procedure [23]. Western blot results were quantified in the Quantity One software (v.4.6.9). The resulting intensity values were then normalized to β-actin. The expression level of the β-actin gene did not change significantly according to our analysis of transcriptomic data. Statistical significance was calculated by the nonparametric Kruskal–Wallis test and the pairwise Wilcoxon test with the Benjamini–Hochberg correction using R package stats (v.4.3.0). Antibodies and dilutions employed in this study were as follows: anti–beta-actin (1:3000; cat. # ab8226, Abcam), anti-MMP9 (1:2000; cat. # ab228402, Abcam), anti-TNFa (1:2000; cat. # MAA133Ra21, CloudClone), anti–α-smooth muscle actin (α-SMA; 1:2000; cat. # ab124964, Abcam), anti-CD68 (1:2000; cat. # ab201340, Abcam), anti-CD163 (1:200; cat. # ab199402, Abcam), anti–E-cadherin (1:3000; cat. # ab76055, Abcam), and anti–IL-10 (1:2000; cat. # ab25073, Abcam) antibodies with respective secondary antibodies: a horseradish peroxidase (HRP)-conjugated goat anti-rabbit IgG (H+L) antibody (1:7000; cat. # AS014, ABclonal, USA) and an HRP-conjugated rabbit anti-mouse IgG (H+L) antibody (1:40000; cat. # ab97046, Abcam, USA). Quantitative densitometric analyses were performed on digitized images of immunoblots in the Quantity One v.4.6.9 software (Bio-Rad, USA).

### 2.9. Statistical analysis

The data were subjected to statistical analysis in the STATISTICA 6.0 software (Statsoft, USA). The Shapiro–Wilk test was carried out to check whether the data distribution was normal. The one-way ANOVA F-test with Newman–Keuls *post hoc* analysis was applied to significant main effects and interactions to evaluate differences between some sets of means.

## 3. Results

### 3.1. Outline of sequencing results

On average, ~37 million paired-end reads (range: 32–50 million) were obtained from each sample by Illumina stranded sequencing (S1 Table). Genome mapping using the STAR software identified 785 million (86.8%) uniquely mapped reads, whereas 39 million (4.3%) reads were mapped more than once.

**Fig 2:**
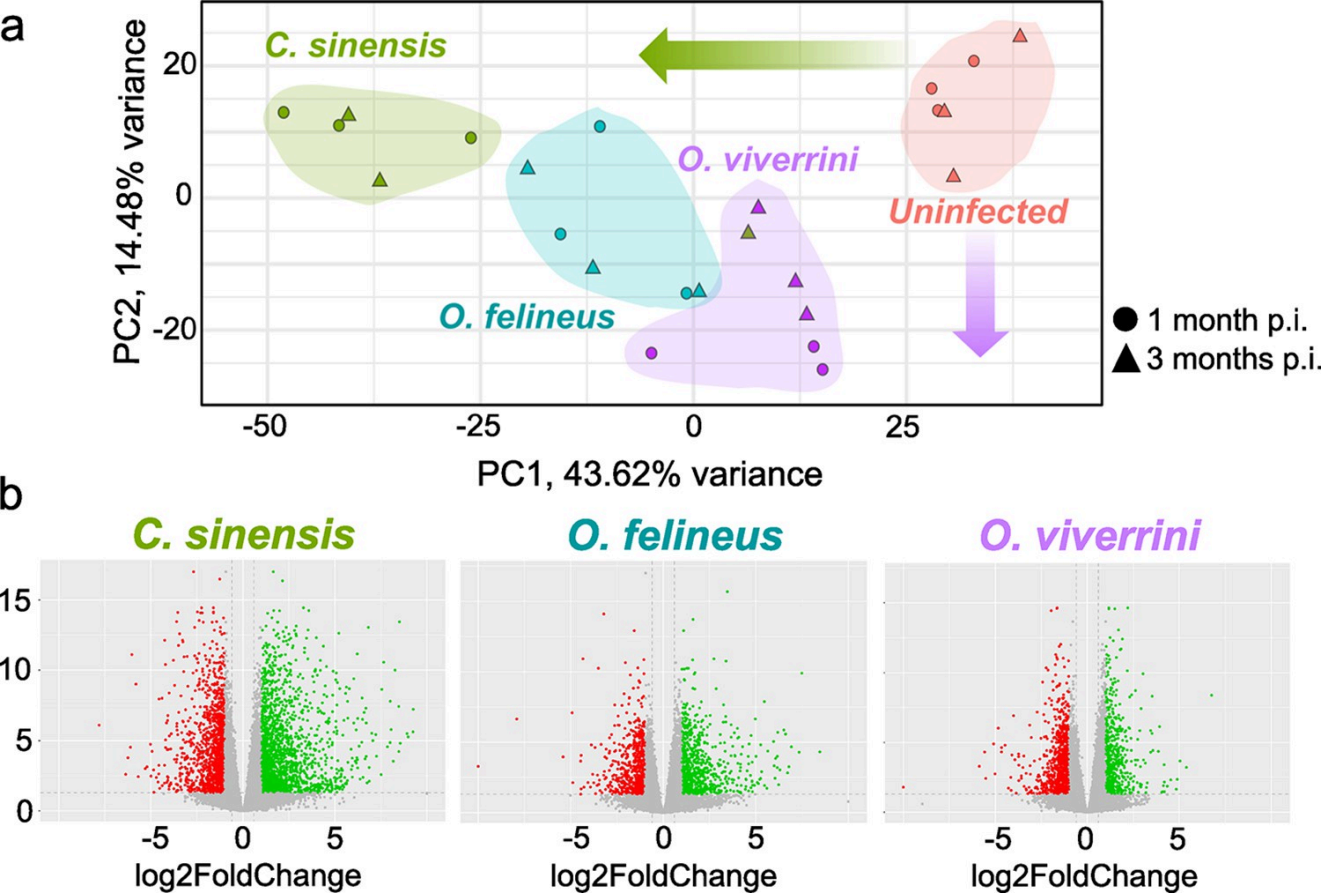
Sequencing data on liver samples from hamsters infected with *O*. *viverrini*, *O*. *felineus*, or *C*. *sinensis* at 1 and 3 months p.i. **a.** PCA of samples; **b.** Volcano plots of the distribution of DEGs (|log_2_FoldChange| > 1) and P_adj_ < 0.05 [-log_10_(P_adj_) > 1.3]. Genes with reduced expression are highlighted in red, and genes with elevated expression are highlighted in green.

PCA revealed clustering of samples into principal components 1 and 2 depending on trematode species (Fig 2A). PC1 reflects the response factor to any infection, and the severity of this response was found to increase toward *C*. *sinensis* infection. PC2 probably indicates some specific response to *O*. *viverrini* infection. Moreover, PCA showed clustering of RNA samples into four separate groups based on liver fluke species. *C*. *sinensis* caused the largest shift in PC1, *O*. *viverrini* the smallest shift in PC1, and infection with *O*. *felineus* occupied an intermediate position. For principal component 2, the largest shift was caused by infection with *O*. *viverrini*, and PC2 did not explain any variation in the group of *C*. *sinensis* samples.

A total of 3148 genes were found to be differentially expressed (P_adj _< 0.05, |log_2_FC| > 1) in *C*. *sinensis*–infected hamsters (1884 upregulated and 1264 downregulated) as compared with the uninfected animals (S2 Table). A total of 1464 genes proved to be differentially expressed in *O*. *viverrini*–infected hamsters as compared with the uninfected animals (593 upregulated and 871 downregulated) (S3 Table), and 1408 genes were differentially expressed in *O*. *felineus*–infected hamsters as compared with the uninfected animals (779 upregulated and 629 downregulated) (S4 Table). Volcano plots of differentially expressed genes (DEGs) between infected and uninfected groups corresponded to one of the time points analyzed (1 or 3 months) are presented in S1 Fig.

### 3.2 Enrichment analysis of DEGs

Functional gene enrichment analysis revealed that the largest number of cellular pathways was associated with infection by *C*. *sinensis*: 329 significantly overrepresented GO biological processes (false discovery rate [FDR] < 0.05), 14 significantly enriched (q-value < 0.05) gene sets (hallmarks) according to the Molecular Signatures Database, and 57 significantly enriched (P_adj_ < 0.05) KEGG pathways. The smallest number of pathways was associated (in terms of significant enrichment) with *O*. *viverrini* infection: 65 significantly overrepresented GO biological processes, eight significantly enriched (q-value < 0.05) MSigDB gene sets (hallmarks), and 25 significantly enriched KEGG pathways. *O*. *felineus* infection was associated with 211 significantly over-represented GO biological processes, 15 significantly enriched MSigDB gene sets (hallmarks), and 30 significantly enriched KEGG pathways.

**Fig 3:**
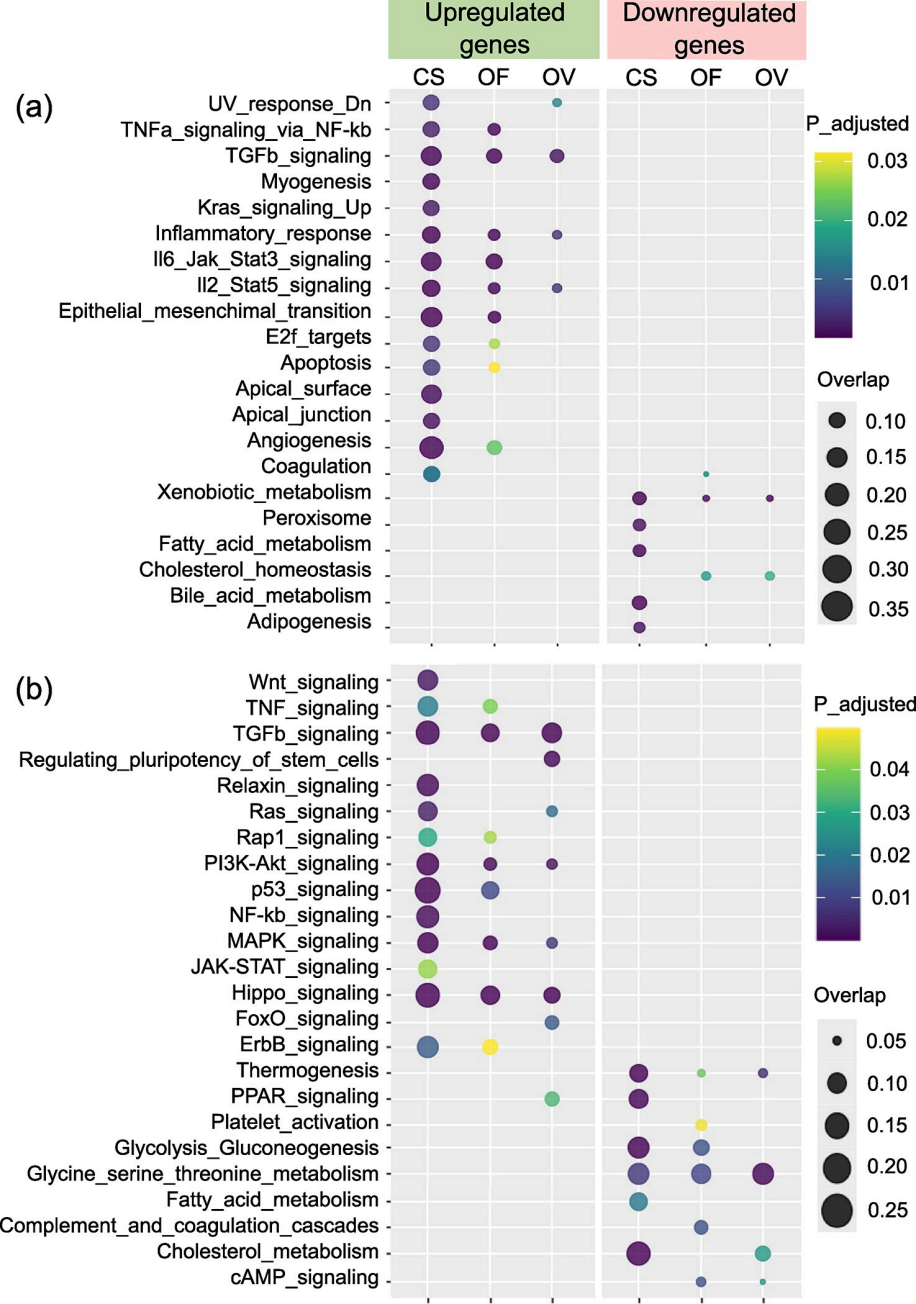
**Enrichment analysis of DEGs using MSigDB gene sets (a) and KEGG signaling pathways (b).** CS: *C*. *sinensis*, OF: *O*. *felineus*, OV: *O*. *viverrini*. The size of the dots indicates the Overlap score of the pathway genes, and the color of the dots reflects different q-values (a) and P_adj_ values (b).

Fig 3A presents 23 significantly enriched gene sets (hallmarks) (q-value < 0.05) according to the Molecular Signatures Database. The MSigDB gene sets most enriched within the group of overexpressed genes—common to all three infections—were the inflammatory response, IL2-STAT5 signaling pathway, and TGF-beta signaling pathway (Fig 3A). Xenobiotic metabolism turned out to be enriched within the group of downregulated genes common to all three infections. Of note, enriched pathways common for *O*. *viverrini* and *C*. *sinensis* were found that were not affected in *O*. *felineus*–infected animals, in particular, genes downregulated in response to ultraviolet radiation. For *O*. *felineus* and *C*. *sinensis* infections, but not for *O*. *viverrini*–infected animals, pathways associated with inflammation were detected, in particular TNF-α signaling via NF-kB, IL6-JAK-STAT3 signaling, epithelial-mesenchymal transition, apoptosis, E2F targets, and angiogenesis. The MSigDB gene sets significantly associated only with *C*. *sinensis* infection were Hallmark_KRAS_signaling_up (M5953, genes upregulated by KRAS activation), Hallmark_apical_surface (M5916, genes encoding proteins over-represented on the apical surface), Hallmark_myogenesis (M5909), Hallmark_G2M_checkpoint (M5901), and Hallmark_apical_junction (M5915, components of the apical junction complex).

Fig 3B presents 32 most significantly enriched KEGG signaling pathways. The KEGG signaling pathways common to the three infections in the subgroup of upregulated DEGs were TGF-beta and PI3K-Akt signaling pathways and Hippo and MAPK signaling. The *O*. *viverrini* infection subgroup of DEGs was found to be significantly enriched with signaling pathways regulating pluripotency of stem cells (Fig 3B). *C*. *sinensis* infection stimulated Wnt, Relaxin signaling, NF-kB signaling, and JAK-STAT signaling. Pathways common between *O*. *viverrini* and *C*. *sinensis* infections were found that were not upregulated in *O*. *felineus*–infected animals, in particular, RAS and FOXO signaling pathways. *O*. *felineus* and *C*. *sinensis* infections, but not *O*. *viverrini* infection, were associated with pathways related to high levels of inflammation, such as TNFα, chemokine, and P53-signaling pathways, Rap1 and ErbB signaling.

### 3.3. Gene cluster analysis of DEGs

**Fig 4:**
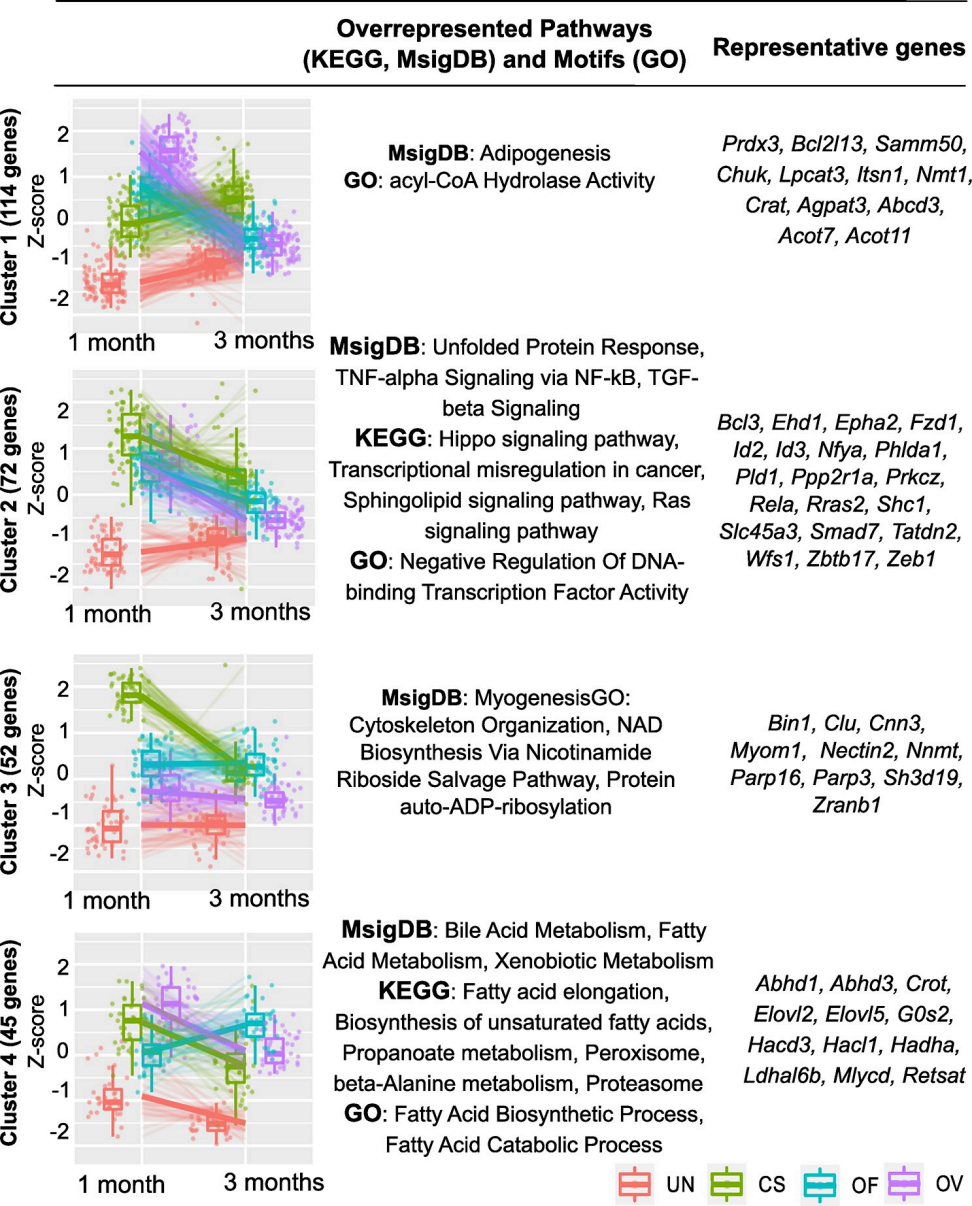
Clustering analysis of DEGs found in the liver of hamsters (*M*. *auratus)* infected with one of three species of trematodes (at 1 and 3 months p.i.). Likelihood ratio (LRT) test revealed gene clusters exhibiting particular patterns across samples. Pathway enrichment analysis was performed using Gene Ontology (GO), Kyoto Encyclopedia of Genes and Genomes (KEGG), and Molecular Signatures Database (MsigDB) databases. UN: Uninfected; CS: *C*. *sinensis*, OF: *O*. *felineus*, OV: *O*. *viverrini*.

In-depth clustering analysis revealed specific patterns of DEGs across the groups of liver RNA samples. To identify such genes, we performed the likelihood ratio test, which grouped genes based on their expression patterns across groups of liver RNA samples. Thus, the genes were subdivided into several clusters (gene groups) (Figs 4, S2 and S5 Tables). In particular, cluster 1 (DEGs upregulated in *O*. *viverrini* and *O*. *felineus* but not in *C*. *sinensis* at 1 month p.i.: (114 genes). Cluster 2 (DEGs upregulated in all three trematode infections at 1 month p.i.: 72 genes); cluster 3 (DEGs upregulated only in *C*. *sinensis*–infected animals at 1 month p.i.: 52 genes); and cluster 4 (DEGs upregulated only in *O*. *viverrini*–infected and *C*. *sinensis*–infected animals at 1 months p.i.: 35 genes).

MsigDB pathways that were found to be enriched (FDR < 0.05) within cluster 1 (upregulated at 1 month p.i.) are related to adipogenesis, and Acyl-CoA Hydrolase Activity was an over-represented GO term. The KEGG pathways most enriched within cluster 2 included Ras and Hippo signaling, transcriptional misregulation in cancer, and sphingolipid signaling pathway (Fig 2). MsigDB pathways that were found to be enriched (FDR < 0.05) within this cluster are related to TNF-α signaling via NF-kB and TGF-beta signaling pathway.

In contrast to cluster 2, the MsigDB pathway that was found to be enriched (FDR < 0.05) within cluster 3 (upregulated in *C*. *sinensis* only at 1 month p.i.) was myogenesis. It was this module that contained 52 genes whose products are associated with cytoskeleton organisation, NAD biosynthesis via nicotinamide riboside salvage pathway, and protein auto-ADP-ribosylation.

The KEGG pathways most enriched within cluster 4 (upregulated in *O*. *viverrini*–infected and *C*. *sinensis*–infected hamsters) included fatty acid elongation, biosynthesis of unsaturated fatty acids, propanoate metabolism, and peroxisome proteasome (Fig 2). MsigDB pathways that proved to be enriched (FDR < 0.05) within this cluster are related to bile acid metabolism, fatty acid metabolism, and xenobiotic metabolism.

### 3.4. Estimation of liver cell type proportions in bulk expression

Cell heterogeneity is of interest in profiling changes in tissue composition associated with pre-cancerous changes. Measures of cell composition can be leveraged to identify cell-specific differential expression from bulk RNAseq data.

Each cell type has its own expression profile and metabolic and signaling pathways and responds to an infectious agent (such as a helminth) in a unique way. On the other hand, it is unknown which pathways in liver cells in general and the responses of which cell types in particular may explain the difference in the carcinogenic properties among the liver flukes. Using the bisqueRNA package for R, interspecies differences in the response of different liver cell types to the trematode infections were determined next.

The bisqueRNA package extracts trends in cellular composition from normalized RNA-seq data by means of only cell type–specific marker genes that have been obtained using single-cell reference data [21]. The logic behind this approach is that the expression of marker genes will directly correlate with the proportion of a relevant cell type, that is, the higher the expression of marker genes, the greater is the contribution of the cell type that owns this marker. This linear covariance can be captured in PCA. Moreover, the more specific a gene’s expression to a given cell type, the more accurately its expression will reflect the contribution of this cell type. The first component from PCA has been shown to capture most of the variance and can provide an estimate of the differences in the response of different cell types to a factor of interest [20].

Using a single-cell–based reference profile [21], 20 cell types were identified using sequencing of 8444 cells and lists of marker genes for each cell type. As a result of applying this package to our transcriptome data, it was revealed that there were significant differences for cholangiocytes, stellate cells, macrophages, endothelial cells, and hepatocytes (S6 Table).

**Fig 5:**
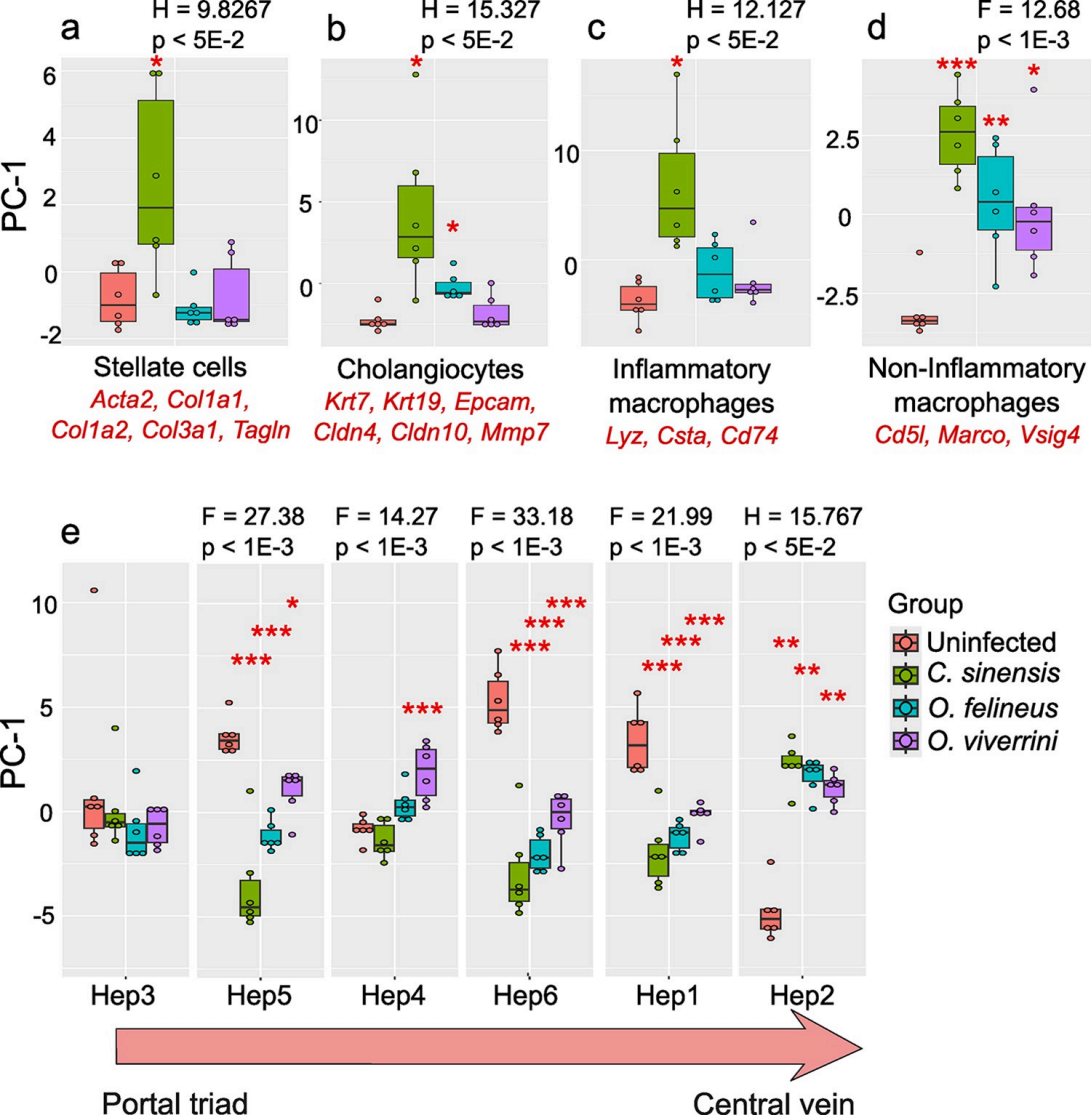
Cell-proportion estimates in RNAseq data. The figure shows proportion estimates for stellate cells (a), cholangiocytes (b), inflammatory macrophages (c), noninflammatory macrophages (d), and hepatocytes of six cell types (e). The arrow indicates the order of arrangement of hepatocyte types from the portal triad (Hep3) to the central vein (Hep2). The data were analyzed using ANOVA (F) or Kruskal-Wallis (H) tests and are presented as box-and-whisker diagram; *p < 0.05; **p < 0.01; ***p < 0.001 as compared to the uninfected (Un) group.

The contribution of stellate cells—whose markers of the activated form are *Acta2*, *Col1a1*, *Tagln*, *Col1a2*, *Col3a1*, *Sparc*, and *Rbp1*—changed significantly only during infection with *C*. *sinensis* compared to the control (pairwise Kruskal–Wallis test with Benjamini–Hochberg correction, P_adj_ = 0.030) (Fig 5A).

*C*. *sinensis* infection caused the strongest response of cholangiocytes, which are characterized by overexpression of such genes as *Krt7*, *Krt19*, *Epcam*, *Fxyd2*, *Cldn4*, *Cldn10*, *Mmp7*, *Cxcl1*, and *Tff2* (Kruskal–Wallis test with Benjamini–Hochberg correction, P_adj_ = 0.013), whereas no significant changes in cell types were detected during *O*. *viverrini* infection (Fig 5B).

The analysis detected two cell types related to macrophages (Fig 5C and 5D). Both cell populations are positive for the Cd68 marker, with inflammatory macrophages featuring overexpression of genes *Lyz*, *Csta*, and *Cd74*, and the noninflammatory macrophages are distinguished by overexpression of e.g. *Cd5l*, *Marco*, *Vsig4*, *Ccdc88a*, and *Lipa*. Inflammatory macrophages produce proinflammatory cytokines (IL-1β and TNFα) and are antigen-presenting cells capable of inducing Th1/Th17 cellular responses and killing pathogens. Anti-inflammatory macrophages, on the contrary, produce anti-inflammatory cytokines IL-10, TGF-β, and others and induce Th2/T_reg_ cellular responses. They respond to extracellular parasites and are associated with resolution of inflammation and regeneration of damaged tissue [28].

This method uncovered a significant response of inflammatory macrophages only to *C*. *sinensis* infection (pairwise Kruskal–Wallis test with Benjamini–Hochberg correction, P_adj_ = 0.013) (Fig 5C and S7 Table), whereas the response of anti-inflammatory macrophages changed significantly for all infections compared to uninfected animals (ANOVA with Tukey’s correction: for *C*. *sinensis*: P_adj_ = 0.00003, *O*. *felineus*: P_adj_ = 0.006, *O*. *viverrini*: P_adj_ = 0.014) (Fig 5D).

The six hepatocyte types in question [21] are characterized by lower proliferative activity and overexpression of albumin (ALB). The difference between hepatocytes that separates them into different cell types is the location of the cell types in the acinus on the central vein–portal triad axis (Fig 5E). Hep3 cells are periportal hepatocytes, while Hep2 are pericentral hepatocytes. Hep1 cells are located closer to periportal hepatocytes, and hepatocytes of the Hep5, Hep4, and Hep6 types are located between Hep3 and Hep1 cells (Fig 5E).

Periportal hepatocytes (Hep3) are characterized by the expression of such marker genes as *Hmgcs1*, *Acss2*, *Tm7sf2*, *Tmem97*, *Msmo1*, and *Lepr*, and pericentral hepatocytes (Hep2) by the expression of *Ghr*, *Aldh6a1*, *Rcan1*, *Ar*, *Plin1*, *Rprd1b*, and others.

Liver fluke infections induced changes in the proportion of pericentral Hep2 cells and proportions of Hep1, Hep5, and Hep6 central hepatocytes but no significant changes were detected in the proportion of periportal Hep3 hepatocytes (Fig 5E). Full statistical comparisons between hepatocyte clusters by ANOVA with Tukey’s test and by the pairwise Kruskal–Wallis test with the Benjamini–Hochberg correction are presented in S7 Table.

### 3.5. Validation of protein expression in the liver

**Fig 6:**
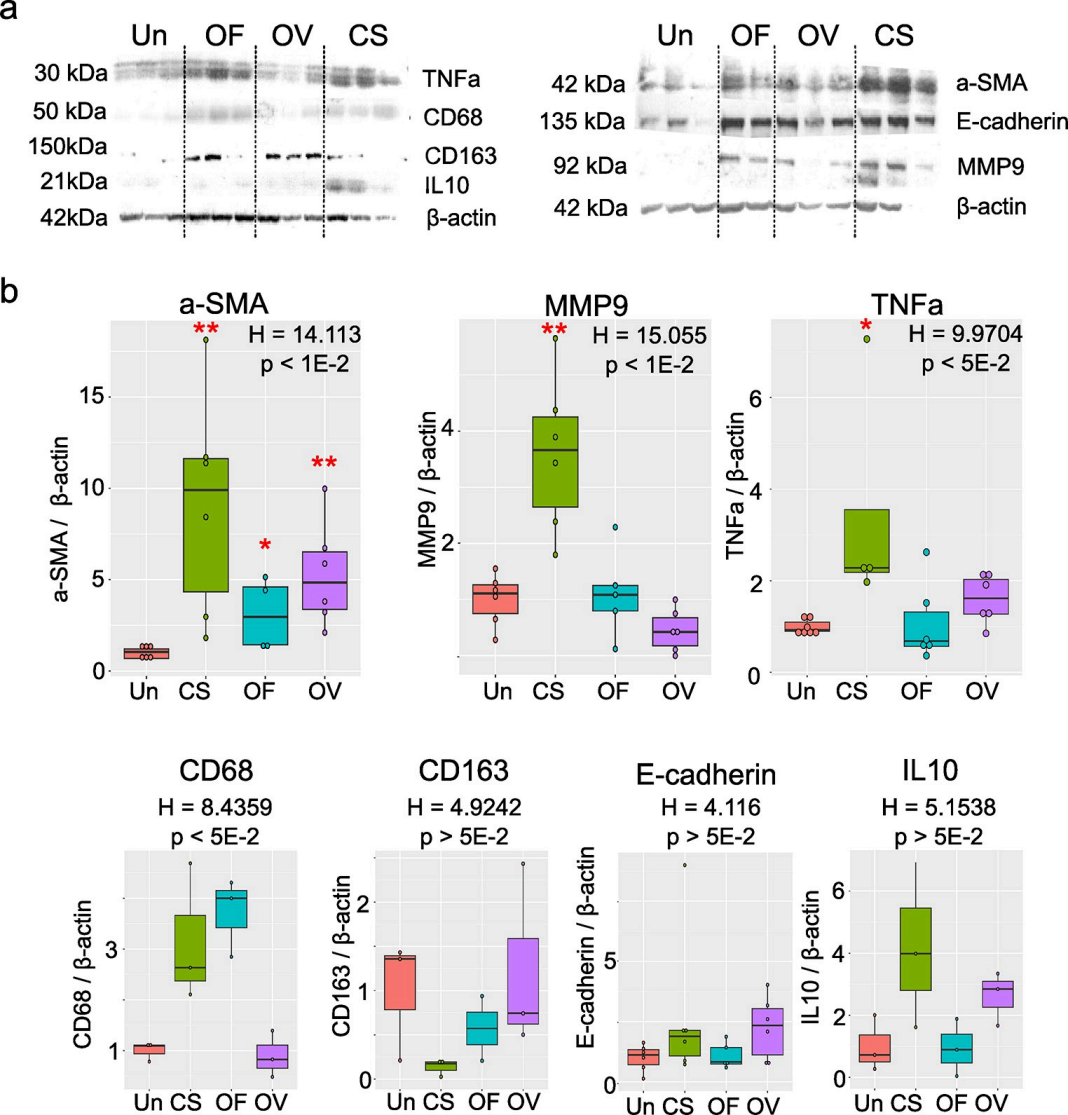
Levels of marker proteins in the liver of hamsters (*M*. *auratus)* with *O*. *felineus* (OF), *O*. *viverrini (*OV), or *C*. *sinensis* (CS) infection at 3 months p.i. as assessed by western blotting. **a**. Immunoblots. Representative images are shown. α-SMA: α-smooth muscle actin. **b**. Results of the densitometry of the immunoblots (A); they are normalized to β-actin. The data are presented as box-and-whisker diagram; *p < 0.05; **p < 0.01 as compared to the uninfected (Un) group (Kruskal-Wallis test).

Western blot analysis of marker proteins was performed on hamsters’ liver lysates at 3 months p.i. (Fig 6A and 6B). α-SMA levels were significantly increased in liver tissue in all three infections (P = 0.0065 for *C*. *sinensis* infection, P = 0.0229 for *O*. *felineus* infection, and P = 0.0065 for *O*. *viverrini* infection), thus confirming activation of stellate cells and the development of fibrosis in liver tissue. Matrix metalloproteinase MMP9 was also found to be upregulated in *C*. *sinensis* infection (P = 0.026), and this finding may be related to epithelial–mesenchymal transition, which was found above to be associated with this infection in our functional enrichment analysis of DEGs. CD68, which is a macrophage marker protein, was upregulated by *C*. *sinensis* infection and *O*. *felineus* infection. Levels of IL10, CD163, and E-cadherin did not change significantly at 3 months p.i.

### 3.6. Liver histopathology

**Fig 7:**
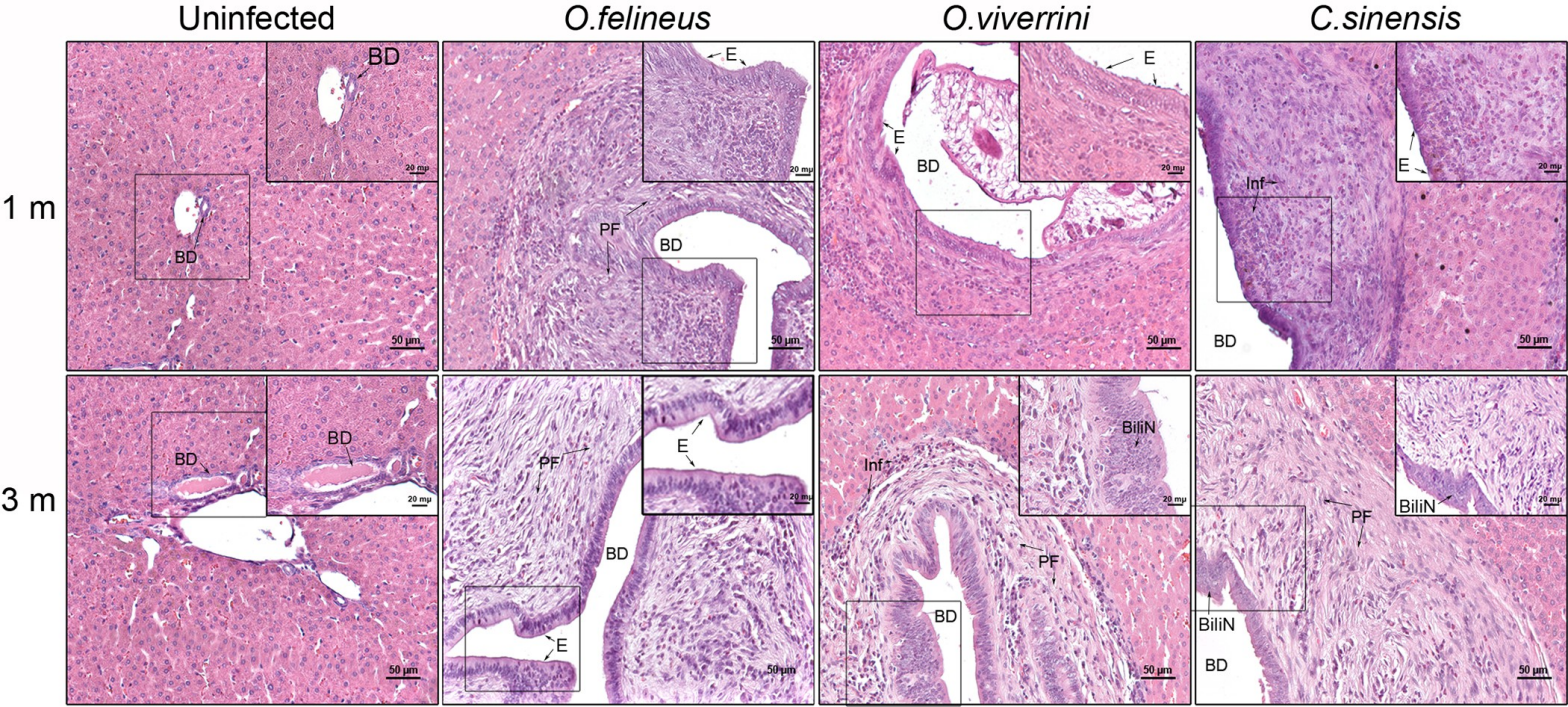
Liver histopathology of hamsters uninfected or infected with *C*. *sinensis*, *O*. *felineus*, or *O*. *viverrini* at 1 and 3 months p.i. BD: bile duct, PF: periductal fibrosis; E: epithelium of bile duct; BiliN: biliary intraepithelial neoplasia; Inf: inflammatory infiltrate. Scale bar = 50 μm in main images, and scale bar = 20 μm in insets.

A scoring method of the ratio (morphometry) type [26] was applied to structural liver lesions (S8 Table). The most common structural liver lesion was periductal fibrosis, which was registered in all infections at 1 month p.i. but was most severe in *C*. *sinensis*–infected and *O*. *felineus*–infected hamsters (Fig 7).

**Fig 8:**
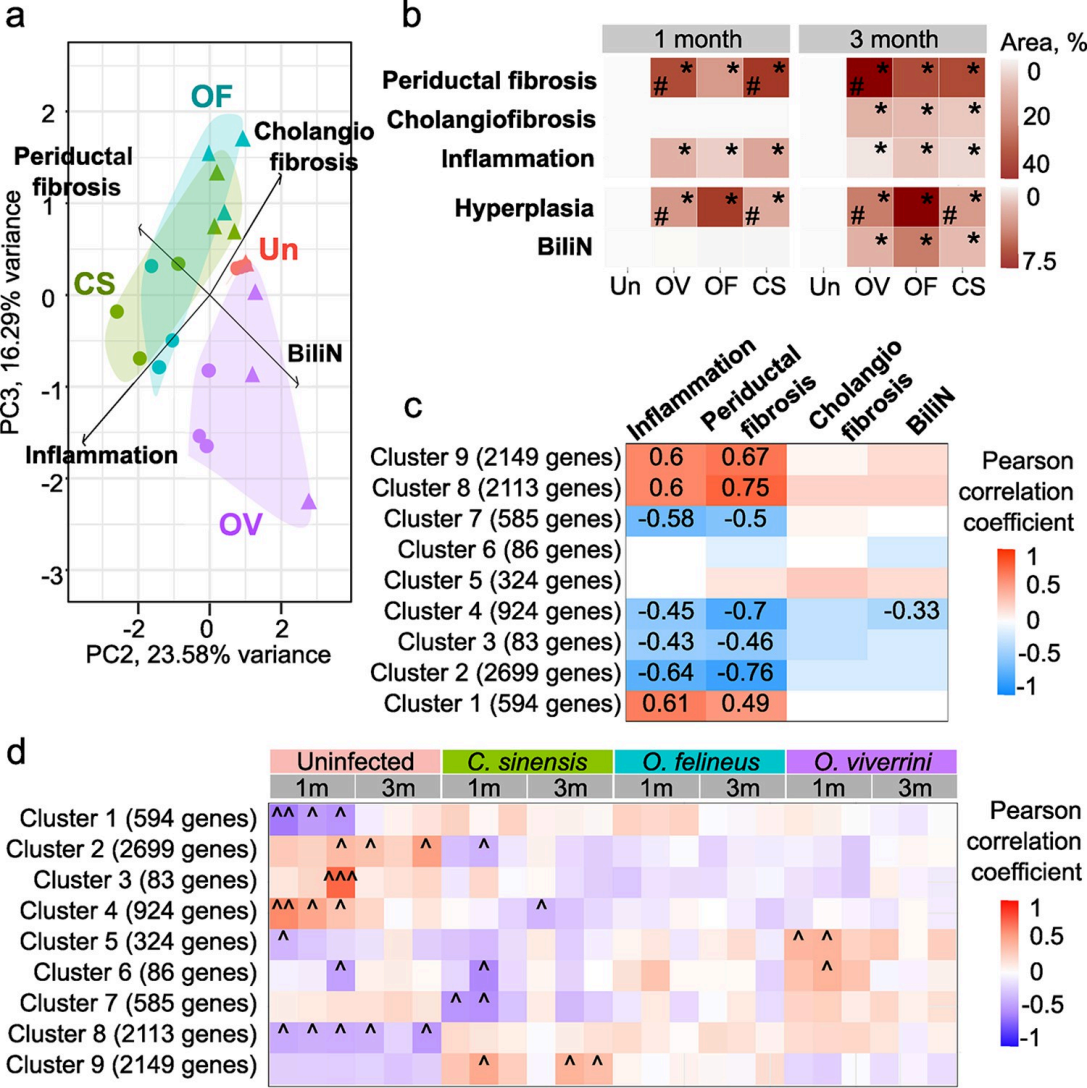
Quantitative histological data and association with coexpressed gene clusters in the liver. a. PCA based on semiquantitative histological evaluation. A scoring method of the ratio (morphometry) type [26] was applied to data from measurements (S8 Table). This method is based on examination of several fields of view in a tissue (e.g., 10 random 400× fields) for each animal, each field was scored, and a mean score was assigned to the whole tissue of that animal. Each slice of a lobe was analyzed in all fields of view (20–30 fields). Each field of view was divided into 100 squares in Morphometry software. PC: principal component; **b.** Heatmap of liver morphometry data. Color codes reflect the area of structural damage as a percentage of the total area. *p < 0.05 as compared to the uninfected animals, ^#^p < 0.05 as compared to *O*. *viverrini*. Un: uninfected; OF: *Opisthorchis felineus*, OV: *O*. *viverrini*, CS: *C*. *sinensis*; BiliN: biliary intraepithelial neoplasia. **c.** Correlation of coexpressed gene modules with structural liver damage (Pearson correlation coefficients greater than 0.3 are shown); **d**. Correlation of coexpressed gene modules with helminth species (^Pearson coefficient > 0.3; ^^Pearson coefficient > 0.5; ^^^Pearson coefficient > 0.7).

Based on the obtained morphometry data, PCA was performed (Fig 8A), and its findings were presented as a heat map (Fig 8B and S8 Table). The area of cholangiofibrosis enlarged significantly only at 3 months p.i. The area of inflammatory-cell infiltration and bile duct epithelial hyperplasia increased at 1 month p.i. and remained elevated at 3 months p.i. in all infected animals. Notably, epithelial hyperplasia was most pronounced during *O*. *viverrini* infection (Fig 8B). Biliary intraepithelial neoplasia (BiliN) increased significantly in all three types of infection at 3 months p.i.

PCA (Fig 8A) revealed that the clustering of tissue samples from *O*. *viverrini*–infected animals was due to BiliN. Samples from animals infected with *C*. *sinensis* and those infected with *O*. *felineus* clustered together, and their clustering was more influenced by periductal fibrosis. It is noteworthy that clustering of tissue samples from animals at 1 month p.i. is associated with inflammation, whereas cholangiofibrosis is characteristic of 3 months p.i.

### 3.7. Weighted gene coexpression network analysis (WGCNA)

This was an analysis of genes whose expression changed significantly during at least one type of infection; nine clusters of coexpressed genes were obtained (Fig 8C and 8D and S9–S17 Tables). Pearson analysis of correlation between the identified clusters of coexpressed genes and the structural liver morphometry data (ST8 Table) revealed cellular signaling pathways associated with some liver lesions (Fig 8C). Pearson correlation coefficients are presented when greater than 0.3.

Inflammation and periductal fibrosis proved to be enhanced in similar ways and were associated with the activation or suppression of similar groups of coexpressed genes (Fig 8C). In particular, cluster 8 (2113 genes) directly correlated with inflammation and periductal fibrosis (Fig 8C and S16 Table). This cluster of genes is enriched with PI3K-AKT-mTOR, TGFβ, and IL6-JAK-STAT3 signaling pathways. Besides, clusters 1 (S9 Table) and 9 (S17 Table) also directly correlate with inflammation and periductal fibrosis and are enriched with signaling pathways such as Hippo, MAPK, ErbB, PI3K-AKT-mTOR, IL6-JAK-STAT3, and TGFβ, consistently with published data [10,11,29,30]. Moreover, an inverse correlation with these structural lesions was observed for clusters 2, 3, 4, and 7 (S10–S12 and S15 Tables). These clusters of genes are enriched with such terms as metabolism of bile acids, fatty acids, and xenobiotics, with the PPAR signaling pathway, and the DNA repair pathway. This outcome may be due to metabolic dysfunction of the liver and bile ducts, which is usually observed in trematodoses.

In contrast, such lesions as cholangiofibrosis and BiliN were associated with a different pattern of gene expression, in particular, gene cluster 1 (TNFα signaling via NF-kB, epithelial-mesenchymal transition, p53 pathway, and TGF-beta signaling) was not correlated with cholangiofibrosis and BiliN. On the other hand, gene cluster 5 (S13 Table) was more associated with cholangiofibrosis and BiliN, but the correlation coefficient was low, just as in cluster 8. Cluster 4, enriched with DNA repair pathway genes, is inversely correlated with BiliN (S12 Table).

Given that we were also interested in differences among the three infections, we examined possible correlations of coexpressed gene modules with the type and duration of infection (Fig 8D). Pearson correlation coefficients are shown when greater than 0.3. Cluster 1 (594 genes, S9 Table), which is enriched with TNFα, NF-kB, TGFβ, p53, and epithelial-mesenchymal transition pathways, inversely correlated with the absence of infection at 1 month p.i. Cluster 7 (585 genes, S15 Table) was inversely correlated with *C*. *sinensis* infection and proved to be enriched with fatty acid, bile acid, and xenobiotic metabolism pathways as well as adipogenesis. Cluster 9 (2149 genes, S17 Table) directly correlated with *C*. *sinensis* infection and proved to be enriched with IL2-STAT5 and IL6-JAK-STAT3 pathways, epithelial-mesenchymal transition, and inflammatory response pathways.

Clusters 5 and 6 are directly correlated with *O*. *viverrini* infection and are enriched with a set of genes regulated by MYC, genes most downregulated in hypoxia-tolerant cancer, and with the pathways of metabolism of fatty acids, bile acids, and xenobiotics as well as oxidative phosphorylation, protein secretion, and others.

Clusters 2 (2699 genes, S10 Table) and 4 (924 genes, S12 Table) directly correlated with the absence of infection and inversely correlated with *C*. *sinensis* infection. Cluster 2 (S10 Table) is enriched with DNA repair pathways, fatty acid and xenobiotic metabolism, and adipogenesis, while cluster 4 is enriched with DNA repair pathways (S12 Table).

## 4. Discussion

This study is the first comparative interspecies analysis of gene expression landscapes in the liver of animals infected with trematodes *O*. *viverrini*, *O*. *felineus*, or *C*. *sinensis*. The integrative approach (comparison of three infections having different carcinogenic properties) seems to be a promising model for investigating the key processes of shifts in the metabolic balance during liver damage and for researching the cascade of regulatory events and dynamics of precancerous changes. Studies on tumors may not answer the question of what underlies helminth-associated biological carcinogenesis. From this point of view, it is most logical to study early events that will subsequently have carcinogenic consequences.

Analysis of the functional enrichment of our group of DEGs uncovered significant enrichment with the inflammatory response and IL2–STAT5, TNF, and TGF-beta signaling pathways in all infected animals. Nevertheless, pathways unique to each infection were found too. For instance, *O*. *viverrini* infection manifested significant association with signaling pathways regulating pluripotency of stem cells. For *O*. *viverrini* and *C*. *sinensis* infections, common pathways were found that were unaffected in *O*. *felineus* infection, in particular, RAS and FOXO signaling pathways. *O*. *felineus* and *C*. *sinensis* infections, but not *O*. *viverrini* infection, were associated with pathways related to high levels of inflammation, such as TNF, chemokine and P53-signaling pathways and Rap1 and ErbB signaling. The enrichment of DEGs with PPAR signaling pathway was associated with *C*. *sinensis* and *O*. *viverrini* infections, but with different directions. PPAR signaling is closely associated with bile acid metabolism, gut microbiota and hepatocyte metabolism. The decreased activity of this pathway in *C*. *sinensis* infection may be related to chronic imbalance in hepatocyte metabolism due to advanced fibrosis. Activation of the PPAR pathway in *O*. *viverrini* infection is associated with alleviated inflammatory signaling.

In addition, estimates of proportions of various affected liver cells revealed some specific responses of individual cell types (stellate cells, cholangiocytes, hepatocytes, and macrophages). The strongest cell response was registered in *C*. *sinensis* infection, including elevated proportions of proinflammatory macrophages and stellate cells, as confirmed by western blot analysis of marker proteins. An anti-inflammatory macrophagic response was observed during infection with each fluke species. On the one hand, our analysis reflects a quantitative contribution of the transcriptome of any cell type; on the other hand, it is unknown whether the contribution is due to the proliferation of a given cell type after infection onset and/or due to an increase in the expression of marker genes.

Moreover, the nature and degree of the severity of liver lesions were shown to be species-specific. Infections with *C*. *sinensis* and *O*. *felineus* mostly featured the development of periductal fibrosis that was accompanied by a decline in the activity of various metabolic pathways in the liver. *O*. *viverrini* infection is characterized by severer hyperplasia and BiliN lesions, and the latter denote a precancerous state. Thus, the related foodborne trematodes have species-specific effects on the liver during infection by causing changes in the expression of various genes and activation or suppression of cellular pathways thereby leading to differences in the severity of structural damage to the liver and possibly contributing to the difference in carcinogenic potential among the trematodes.

Indirectly, the different carcinogenicity of these three liver flukes is confirmed by data on cholangiocarcinoma incidence in endemic areas. Regarding *O*. *viverrini*, in prospective case–control studies, it has been demonstrated that 0.5–3.5% of patients with opisthorchiasis develop cholangiocarcinoma [31,32]. A case–control study indicates that *C*. *sinensis* infection is significantly associated with increased risk of cholangiocarcinoma (OR = 7.3) [4]. According to data from 2016, liver cancer in Korea ranked 5^th^ in the list of the most common types of cancer [33]. Cancer of the liver and of intrahepatic bile ducts in Russia, according to data for 2017, occupied the 18^th^ place in the list of all causes of cancer [reviewed by 1]. Accordingly, the incidence of cholangiocarcinoma is significantly different between Western Siberia and Southeast Asia, which are comparable in terms of the prevalence of liver fluke infection in the population. This observation suggests that carcinogenic potential of *O*. *felineus* is lower than that of *O*. *viverrini* and *C*. *sinensis*. Nonetheless, results of a retrospective analysis of autopsy records and medical records still confirm the association between *O*. *felineus* infection and liver cancer in Western Siberia [34–36].

It is noteworthy that inflammation and periductal fibrosis behaved in a similar way in our study and led to the activation of a group of coexpressed genes (cluster 1) that is enriched with signaling pathways such as Hippo, MAPK, ErbB, PI3K-AKT-mTOR, IL6-JAK-STAT3, and TGFβ, overall consistently with published data. For example, TNF (tumor necrosis factor α) triggers specific NF-κB signaling pathways leading to the activation of an inflammatory response [30,37]. The latter pathology is characteristic of every chronic liver disease, including nonalcoholic fatty liver disease, viral hepatitis, and primary biliary cholangitis [37]. Sustained activation of NF-κB by TNF causes stimulation of cancer cell proliferation, prevents apoptosis during drug resistance, and enhances angiogenesis and tumor metastasis [38]. The PI3K–Akt signaling pathway participates in a variety of biological processes, including stimulation of cell proliferation, inhibition of apoptosis, regulation of tissue inflammation, and tumor growth and invasion [39]. The Hippo pathway in the liver is a key mediator of its development and regeneration, including the activation of proteins YAP and TAZ, which control cell proliferation and migration, epithelial-mesenchymal transition, and organization of the extracellular matrix [40]. MAPK signaling pathways are launched in response to external triggers, such as growth factors, mitogens, cytokines, and stress, and influence cell proliferation, differentiation, apoptosis, inflammation, and metabolism through activation of ERK, Ras/Raf1, p38, and stress-activated protein kinases SAPK or JNK [41].

Some of the cluster 1 pathways characteristic of inflammation and periductal fibrosis were associated differently with the three infections. In particular, in our group of DEG genes, epithelial–mesenchymal transition and the IL6–JAK–STAT3 signaling pathway were associated with *C*. *sinensis* and *O*. *felineus* infections but not with *O*. *viverrini* infection. These data are also consistent with the histological finding that inflammation and periductal fibrosis were less likely in *O*. *viverrini* infection than in the other two parasitoses. The IL6–JAK–STAT3 signaling pathway is hyperactivated in patients with chronic inflammation [42–44]. Activation of epithelial–mesenchymal transition and extracellular-matrix–remodeling proteins has been previously demonstrated in various liver fluke infections [2,10,12,45] and is closely related to the TGF-β signaling pathway and activation of stellate cells [18,29]. These cells during inflammation and fibrogenesis have proliferative, migratory, and invasive abilities and are able to stimulate fibrogenesis by deposition of extracellular-matrix molecules [46,47].

It is worth mentioning that clusters 1, 8, and 9 of coexpressed genes, which are related to inflammation and periductal fibrosis, were not significantly associated with cholangiofibrosis and BiliN, implying the involvement of other factors or genes necessary for the development of these lesions. In particular, there were differences between inflammation and BiliN in cluster 5, which contains a subgroup of genes regulated by MYC, genes most downregulated in hypoxia-tolerant cancer, and pathways of oxidative phosphorylation and protein secretion. For cluster 5, the magnitude of correlation is more significant with *O*. *viverrini* infection, and this cluster seems to share similarities with this type of infection.

The FOXO pathway in combination with hypoxia is linked with tumorigenesis and angiogenesis [48], in agreement with the more pronounced carcinogenicity of *O*. *viverrini* and *C*. *sinensis* compared to *O*. *felineus*. Accordingly, this pathway was not enriched within the group of DEGs of *O*. *felineus* infection. The FOXO signaling pathway is activated in response to growth factors, insulin, oxidative stress, and hypoxia and is capable of controlling several specific gene expression programs, in particular those suppressing tumor growth through induction of apoptosis genes and cell cycle arrest [48].

Interesting data were obtained in our work during the estimation of proportions of periportal and pericentral hepatocytes in the response to the three infections; these findings are consistent with published data [49]. For instance, periportal hepatocytes normally have an enhanced capacity for gluconeogenesis and express APC: a negative regulator of the HIF and Wnt/β-catenin signaling pathway, which prevents cells from acquiring the pericentral hepatocyte phenotype. After pathological stimuli, there is a shift of periportal hepatocytes toward pericentral hepatocytes [49]. Pericentral hepatocytes have high carcinogenic potential because they are capable of Wnt/β-catenin signaling and produce large amounts of xenobiotic detoxification enzymes, making their zone a site of oxidative stress [50]. It is known that many diseases start developing in the pericentral region of hepatic acini, e.g., drug and alcohol hepatotoxicity, fibrosis caused by parasitosis, nonalcoholic fatty liver disease, steatohepatitis, and cirrhosis [49], overall in line with our data on the significant elevation of pericentral hepatocytes’ proportion in the response to infection.

Thus, various factors take part in the formation of cancer associated with helminth infection, although the exact mechanisms are still debated. Chronic inflammation has been proposed as a key pathway for cancer initiation and progression. Nonetheless, the presence of chronic inflammation alone is not a sufficient condition and does not explain why these three trematode species differ in carcinogenicity. Moreover, in our work, the severest inflammation was found in *C*. *sinensis*–infected and *O*. *felineus*–infected hamsters, not in *O*. *viverrini*–infected ones, even though *O*. *viverrini*, according to indirect data, is considered the most carcinogenic.

## 5. Conclusion

Various studies on mechanisms of the biological carcinogenesis associated with helminth infection have highlighted mechanical injuries by parasites, long-lasting immune-system–mediated pathogenesis, activation of oncogenes, inactivation of tumor suppressor genes, and somatic mutations as key factors. Moreover, in the biliary system, environmental pathogens or exotic microbes that are resistant to host inflammatory responses may contribute to carcinogenesis, might be responsible for enlarged inflammatory and fibrotic responses in cancers, and can facilitate the carcinogenicity [51–53]. An additional mechanism increasing genomic instability at a site of inflammation may be helminth-derived reactive oxysterol-like metabolites. Furthermore, helminth-derived proteins and growth factors promoting host tissue repair and angiogenesis [45,54,55] can also facilitate the proliferation of cells subjected to carcinogenesis initiation. Consequently, all this makes it difficult to directly compare the pathogenesis of several helminth infections in order to identify key factors of the carcinogenicity.

Although a major determinant of the carcinogenic outcome of food-borne trematode infection is unclear, this study provides the first glance at all species-specific pathways that are activated or suppressed in response to such trematodiases. A species-specific response of cellular pathways is still worth checking in more detail in order to identify key factors of the biological carcinogenesis induced by the food-borne trematodes.

## Supporting information

S1 Table Output statistics of raw sequencing data. (XLSX)

S2 Table DEG genes in the *C*. *sinensis*-infected animals (cut-off P_adj_ = 0.05). (XLSX)

S3 Table DEG genes in the *O*. *felineus*-infected animals (cut-off P_adj_ = 0.05). (XLSX)

S4 Table DEG genes in the *O*. *viverrini*-infected animals (cut-off P_adj_ = 0.05). (XLSX)

S5 Table Clustering analysis of DEGs (LRT, the likelihood-ratio test). (XLSX)

S6 Table Gene expression markers for liver single cell clusters. (XLSX)

S7 Table Contributions of individual liver cell types to the response to infection according to ANOVA or Kruskal–Wallis test. (XLSX)

S8 Table Liver histopathology data. (XLSX)

S9 Table Cluster 1 of WGCNA genes and their functional annotation. (XLSX)

S10 Table Cluster 2 of WGCNA genes and their functional annotation. (XLSX)

S11 Table Cluster 3 of WGCNA genes and their functional annotation. (XLSX)

S12 Table Cluster 4 of WGCNA genes and their functional annotation. (XLSX)

S13 Table Cluster 5 of WGCNA genes and their functional annotation. (XLSX)

S14 Table Cluster 6 of WGCNA genes and their functional annotation. (XLSX)

S15 Table Cluster 7 of WGCNA genes and their functional annotation. (XLSX)

S16 Table Cluster 8 of WGCNA genes and their functional annotation. (XLSX)

S17 Table Cluster 9 of WGCNA genes and their functional annotation. (XLSX)

S1 Fig Volcano plots of differentially expressed genes (DEGs) between infected and uninfected groups corresponded to one of the time points analyzed (1 or 3 months). (TIF)

S2 Fig Clustering analysis of DEGs (LRT, the likelihood-ratio test). (TIF)
