# Supplementary figures and images for: Comparative liver transcriptome analysis in hamsters infected with food-borne trematodes Opisthorchis felineus, Opisthorchis viverrini, or Clonorchis sinensis
Source: PLoS Negl Trop Dis. 2024 Dec 9;18(12):e0012685. doi: 10.1371/journal.pntd.0012685 (PMC11627427; doi:10.1371/journal.pntd.0012685)

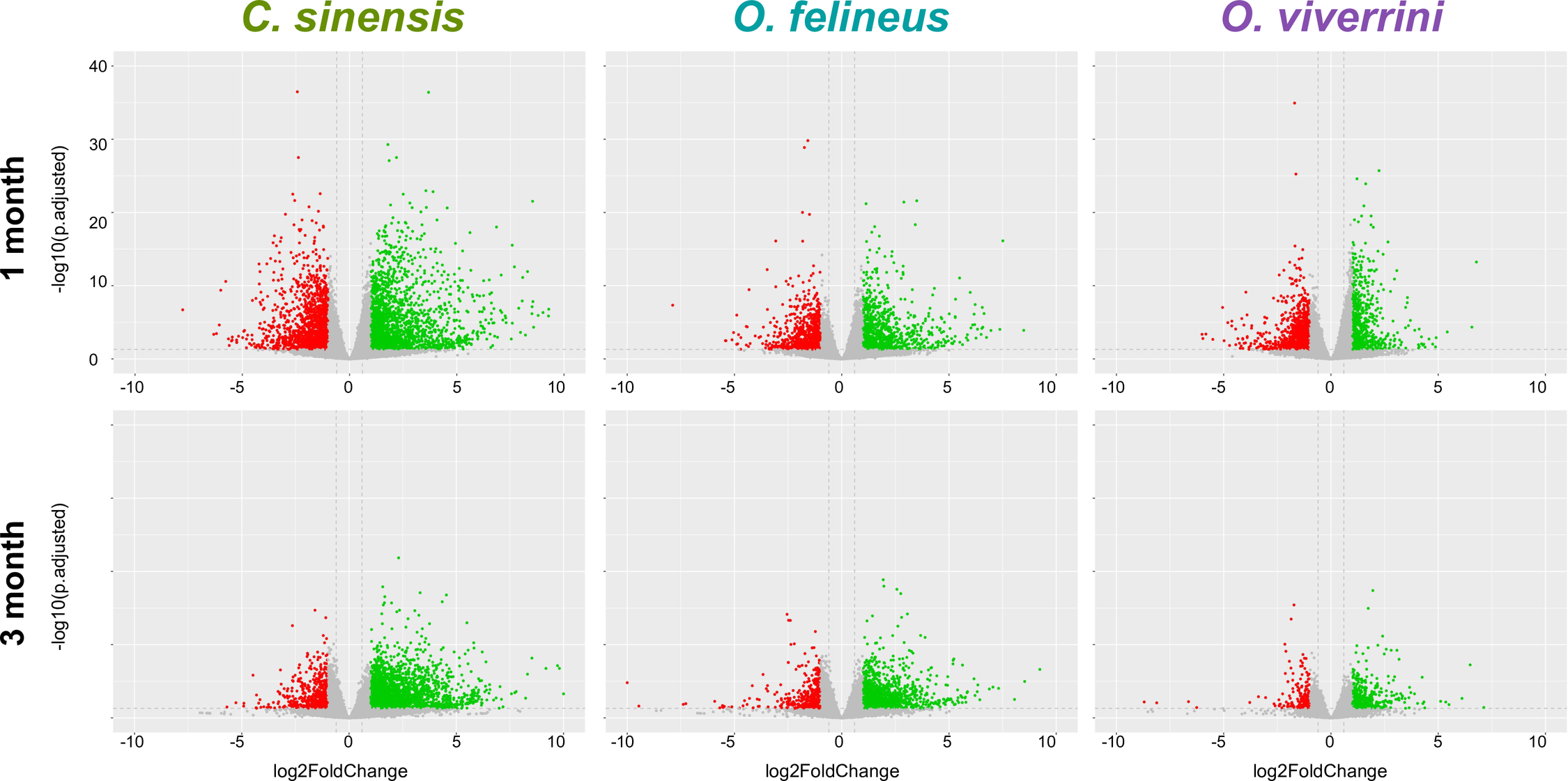

Supplement: S1 Fig — (TIF) [file pntd.0012685.s018.tif]

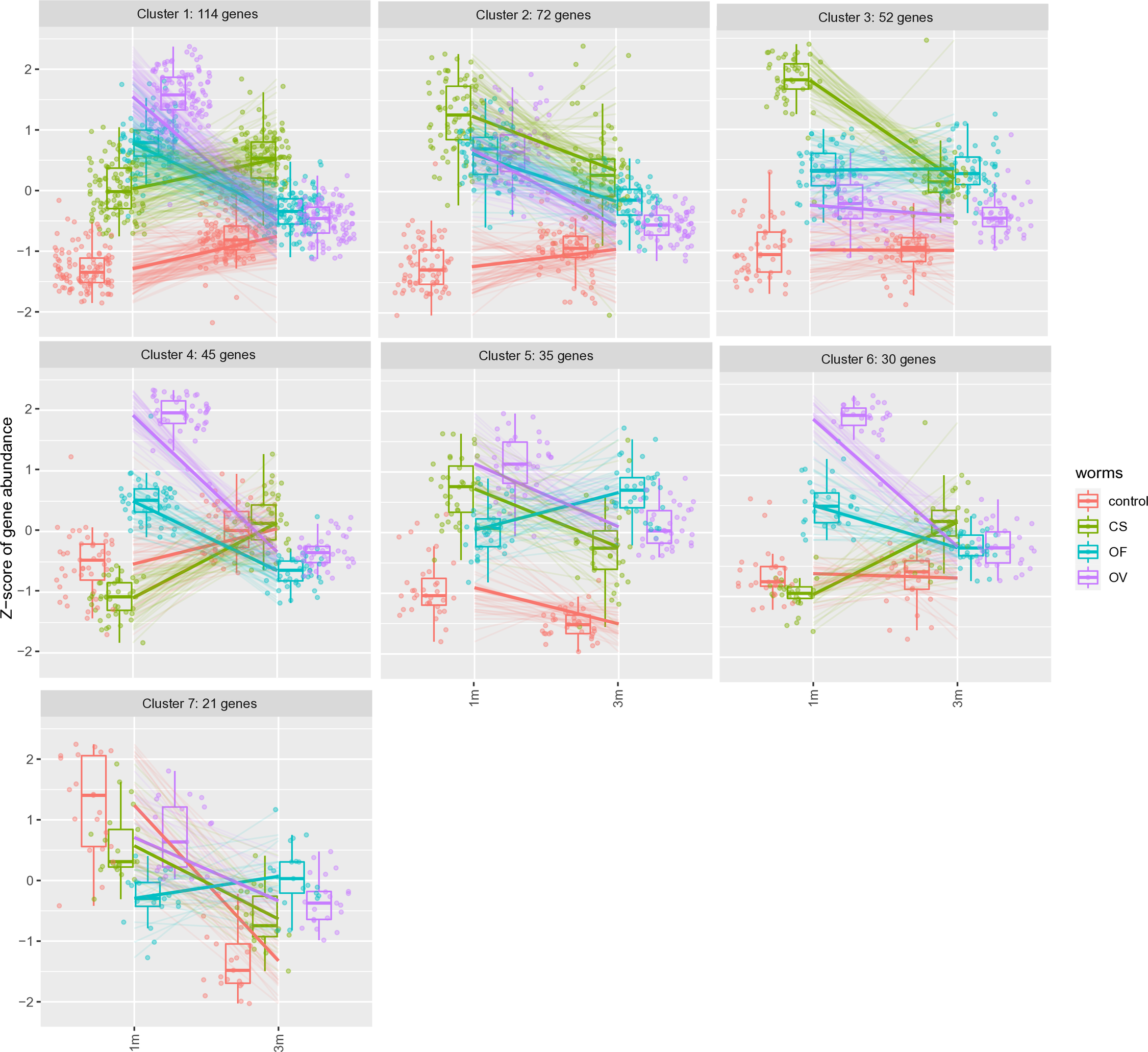

Supplement: S2 Fig — (TIF) [file pntd.0012685.s019.tif]
